# Impact of Omentopexy on Outcomes of Laparoscopic Sleeve Gastrectomy: A Systematic Review and Meta-Analysis of Randomized Controlled Trials

**DOI:** 10.7759/cureus.99511

**Published:** 2025-12-17

**Authors:** Abdelrahman Ibrahim, Swarna Kempe-Gowda, Shivam Bhanderi, Saleh Romman, Chandra Cheruvu

**Affiliations:** 1 Trauma and Orthopaedics, University Hospitals of North Midlands NHS Trust, Stoke-on-Trent, GBR; 2 General Practice, University Hospitals of North Midlands NHS Trust, Stoke-on-Trent, GBR; 3 North Midlands Upper Gastrointestinal (GI) and Bariatric Unit, University Hospitals of North Midlands NHS Trust, Stoke-on-Trent, GBR

**Keywords:** bleeding, laparoscopic sleeve gastrectomy complications, omentopexy, randomized controlled trials (rcts), readmission risk, systematic review and meta-analysis

## Abstract

The routine use of omentopexy to cover the staple line during laparoscopic sleeve gastrectomy (LSG) remains a topic of debate. We aimed to evaluate comparative outcomes of LSG in patients with or without omentopexy. A systematic search of electronic data sources and bibliographic reference lists was conducted. All randomized controlled trials (RCTs) reporting comparative outcomes of LSG in patients with versus without omentopexy were included, and their risk of bias was assessed. Leakage, bleeding, reintervention, gastric torsion, readmission, nausea, vomiting, reflux, operative time, and length of hospital stay were the evaluated outcome parameters. A total of 10 RCTs were included, reporting a total of 1200 patients with (n = 615) and without (n = 585) omentopexy who underwent LSG. Omentopexy was associated with a significantly lower rate of bleeding (odds ratio (OR): 0.37, 95% CI 0.15 to 0.90, P = 0.03), readmission (OR: 0.22, 95% CI 0.08 to 0.57, P = 0.002), and postoperative nausea (OR: 0.35, 95% CI 0.13 to 0.98, P = 0.05) when compared to no omentopexy. No significant difference was found in the rates of staple line leakage (OR: 0.43, 95% CI 0.14 to 1.30, P = 0.13) or reintervention (OR: 0.22, 95% CI 0.05 to 1.04, P = 0.06) between the two groups. The meta-analysis of the best available evidence from RCTs indicates that omentopexy is associated with a reduced risk of bleeding, readmission, and nausea following LSG. The impact of omentopexy on long-term outcomes requires further research.

## Introduction and background

The global prevalence of severe obesity affects a large part of society. According to a WHO report, in 2022, 16% of the adult population suffered from obesity [1]. Bariatric surgery is the most effective treatment [2], and laparoscopic sleeve gastrectomy (LSG) is one of the most widely performed bariatric procedures worldwide [3].

Despite its encouraging anthropometric and metabolic results, concerns remain regarding postoperative complications, most frequently bleeding from the staple line and gastric leakage [4,5]. Bleeding occurs in approximately 2.1% of cases, while gastric leakage is reported in 2.5% [6,7]. These complications lead to significant additional costs, prolonged hospital stays, and increased morbidity [8].

To reduce the incidence of these adverse events, surgeons have proposed technical modifications. Among these, omentopexy has been suggested to cover the staple line and prevent gastric twist, a potential cause of complications. This technique involves suturing the greater omentum to the staple line to provide coverage and mechanical support [9,10].

However, the efficacy of this technique remains controversial. While some studies suggest omentopexy may decrease morbidity and the gastric leak rate [11], other clinical trials have produced conflicting results, and there is currently no consensus on its routine use [2,12].

Therefore, we aimed to conduct an updated systematic review and meta-analysis of the available studies to provide a higher level of evidence concerning the benefits and harms of omentopexy on LSG outcomes.

## Review

Methods

This meta-analysis was conducted in accordance with the standards set by the Preferred Reporting Items for Systematic Review and Meta-Analyses (PRISMA) statement [13]. The methodology, eligibility criteria, and all outcomes investigated were predefined and documented in a review protocol.

Study Design

All randomized controlled trials (RCTs) comparing the outcomes of LSG in patients with and without omentopexy were included. The inclusion criteria were as follows: (i) Population: All adult patients (age > 18 years) of any gender undergoing primary LSG. (ii) Intervention: Patients undergoing LSG with omentopexy. (iii) Comparison: Patients undergoing LSG without omentopexy. (iv) Outcomes: Studies reporting at least one of the primary or secondary outcomes of interest. Primary outcomes included leakage, bleeding, reintervention, and gastric torsion, or the secondary outcomes of reflux, vomiting, nausea, perigastric collection, readmission, wound infection, length of hospital stay (hours), operative time (minutes), and one-year weight loss. (v) Study size: A minimum sample size of 10 patients in each group (at least 20 patients in total).

Studies were excluded if they had not directly compared the two groups, investigated only one group, were non-randomized studies, case reports, case series, reviews, editorials, conference abstracts without sufficient data, biomechanical, cadaveric, or animal studies, or were not available in English.

Search Strategy 

We developed a systematic search methodology (Appendix A) using thesaurus terms, search operators, and database-specific limits. This approach was applied to MEDLINE, Embase, the Cumulative Index to Nursing and Allied Health Literature (CINAHL), the Cochrane Central Register of Controlled Trials (CENTRAL), and Web of Science. Two independent authors conducted this initial search. The authors also evaluated clinical trial registries, including the International Standard Randomized Controlled Trial Number (ISRCTN) registry, ClinicalTrials.gov, and the WHO International Clinical Trials Registry Platform (ICTRP), to find ongoing or unpublished studies. The final search date was April 21, 2025.

Selection of Studies

Two independent reviewers (AI and SK) screened all titles and abstracts found as a result of the search. Where relevance was indicated, the full-texts of relevant articles were obtained and carefully assessed against the predefined eligibility criteria of this review. Studies that met the inclusion criteria were included for the analysis. Any discrepancies arising during this selection phase were resolved through discussion between the two authors. However, if the disagreement persisted, a third author was consulted. Specific care was taken to cross-reference author lists, institutions, and recruitment periods to ensure no overlapping patient cohorts were included, particularly among studies originating from the same region.

**Figure 1:**
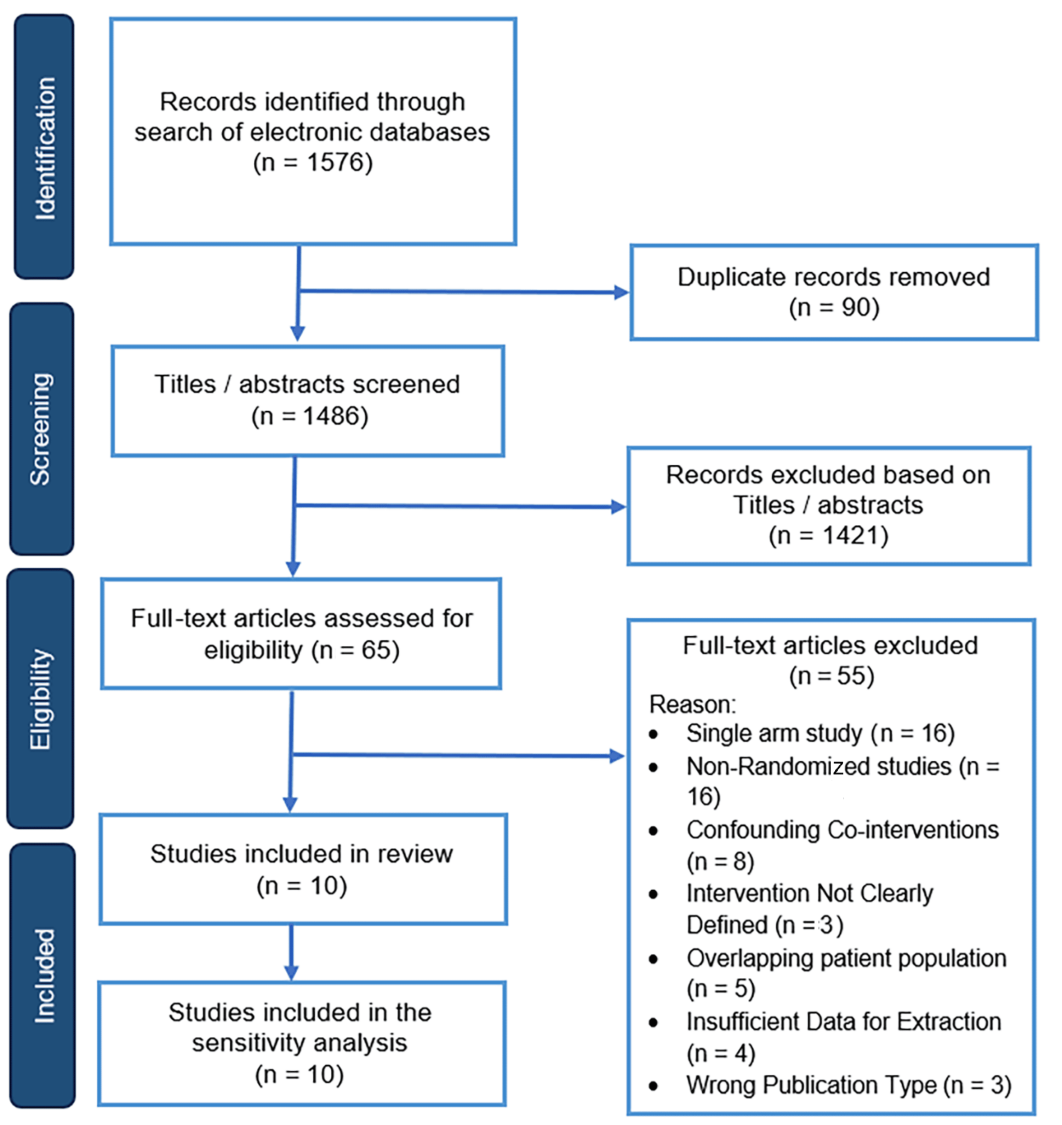
PRISMA flowchart PRISMA: Preferred Reporting Items for Systematic reviews and Meta-Analyses

Following the literature search, 1576 articles were identified. After the removal of 90 duplicates, 1486 articles were screened. Of those, 65 articles were shortlisted for full-text assessment and potential inclusion. After careful evaluation of their full texts, 55 studies were excluded for the following reasons: 16 were single-arm studies, 16 were non-randomized studies, eight had confounding co-interventions, five used an overlapping patient population, four had insufficient data for extraction, three had the wrong publication type, and three did not clearly define the intervention. Therefore, we included 10 comparative studies, all of which were RCTs, enrolling a total of 1200 patients who underwent LSG with either omentopexy (n = 615) or without omentopexy (n = 585) (Figure 1).

Data Extraction and Management

We developed a standardized electronic data extraction Excel sheet (Microsoft Corp., Redmond, USA). This adhered to Cochrane guidance for intervention reviews [14]. Two independent reviewers (AI and SK) extracted all necessary data. This included study characteristics, baseline demographics, and the key clinical outcome data from every included study. Any discrepancies encountered during this process were resolved through discussion with a third author.

Assessment of Risk of Bias

The methodological quality and risk of bias of the 10 included RCTs were assessed independently by two authors (AI and SK). For this, the Cochrane Risk of Bias tool was used [15]. The Cochrane Risk of Bias tool assesses the risk of bias across seven distinct domains: random sequence generation (selection bias), allocation concealment (selection bias), blinding of participants and personnel (performance bias), blinding of outcome assessment (detection bias), incomplete outcome data (attrition bias), selective reporting (reporting bias), and other bias.

The risk of bias regarding random sequence generation was judged to be 'low' in eight studies and 'unclear' in two. Allocation concealment was judged to be at 'low' risk of bias in nine studies and 'unclear' in one. For blinding of participants and personnel, the risk was judged to be 'low' in all 10 studies. However, for blinding of outcome assessment, the risk was 'low' in four studies and 'unclear' in the remaining six. The risk of bias due to incomplete outcome data was 'low' in six studies and 'unclear' in four. Regarding selective reporting, the risk was 'low' in five studies and 'unclear' in five. Finally, the risk of other bias was judged to be 'low' in seven studies and 'unclear' in three. No study was judged to be at 'high risk' of bias in any of the assessed domains.

**Figure 2:**
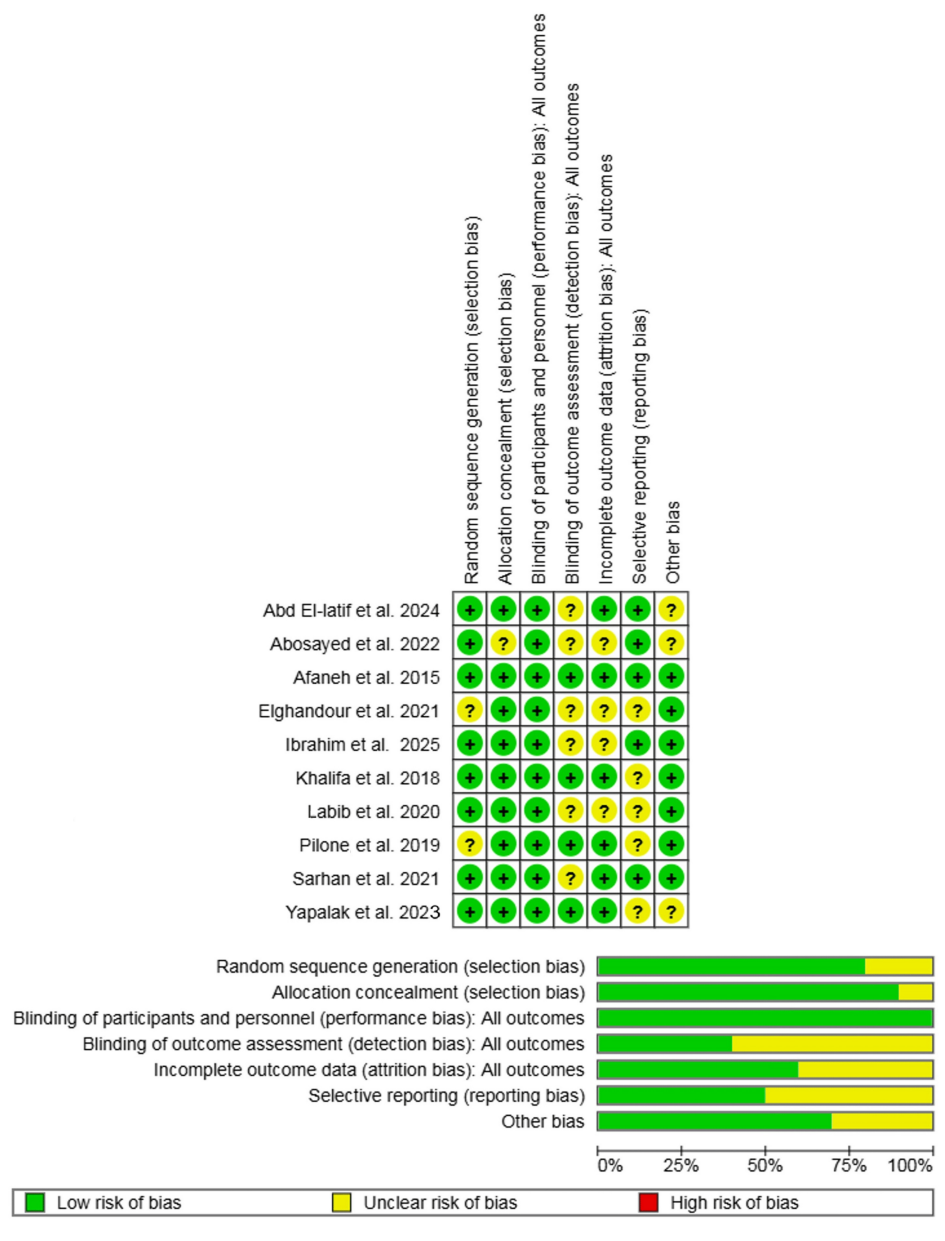
Risk of bias summary and graph showing authors' judgments about each risk of bias item for the RCTs RCTs: randomized controlled trials [16-25]

Any discrepancies identified during this assessment were resolved by discussion between the two authors. If a consensus could not be reached, a third author was consulted for adjudication. The detailed risk of bias assessment is presented in Figure 2.

Summary Measures and Synthesis

We used odds ratio (OR) with a 95% CI as the summary measure for all dichotomous outcomes (e.g., leakage, bleeding). For dichotomous outcomes where studies reported zero events in one or both arms, the Mantel-Haenszel method with continuity correction was utilized. This method was selected to handle sparse data effectively and allow for the calculation of ORs without excluding zero-event trials. As all reported dichotomous outcomes were adverse events, an OR > 1 indicated a higher risk associated with the omentopexy group, thus favoring the non-omentopexy group. Conversely, an OR < 1 would favor the omentopexy group.

All extracted data were initially entered into Review Manager (RevMan) version 7.12.0 (The Cochrane Collaboration, London, UK) by a single reviewer [26]. A second independent reviewer then verified the entered data to confirm its accuracy and completeness. A random-effects model was selected a priori for all analyses to account for the anticipated clinical and methodological heterogeneity inherent in surgical trials (e.g., varying surgeon experience and techniques). Each outcome's results were shown on a forest plot, which included 95% CIs. The level of statistical heterogeneity was assessed using the I² statistic [14], which was interpreted using a scale: 0%-25% suggested possibly unimportant heterogeneity; 26%-75% showed moderate heterogeneity; and 76%-100% represented high heterogeneity. However, we acknowledged that for outcomes with low event rates, random-effects models may underestimate the true level of heterogeneity. Therefore, an I² value of 0% in the presence of sparse data was interpreted with caution, recognizing it may reflect sparse data bias and the use of continuity corrections rather than true homogeneity.

We intended to evaluate publication bias using funnel plots; however, as no outcome included more than 10 studies, funnel plot asymmetry could not be robustly assessed. To verify the robustness of our findings, we performed sensitivity analyses by excluding studies deemed to have a high risk of bias. Additionally, we assessed the influence of each individual study on the overall pooled effect size and heterogeneity by conducting a 'leave-one-out' analysis, sequentially omitting one study at a time.

Results

Demographics

The weighted mean age was 37.5 years in the omentopexy group versus 36.6 years in the non-omentopexy group. The weighted female percentage was 57.6% in the omentopexy group compared to 55.3% in the non-omentopexy group. The weighted mean preoperative body mass index was 45.0 kg/m² in the omentopexy group versus 45.1 kg/m² in the non-omentopexy group. For the entire cohort, the weighted mean follow-up was 8.8 months.

Table 1 presents the study-related data and baseline characteristics of the included populations. 

**Table 1:** Study-related data and baseline characteristics RCT: randomized controlled trial; NR: not reported

Author and Year	Country	Journal	Study Design	Total	No Omentopexy	Omentopexy	Age (years)	Sex (female, %)	Pre-op BMI (kg/m²)	Follow-up
Abd El-latif et al. 2024 [16]	Egypt	The Egyptian Journal of Hospital Medicine	RCT	64	32	32	41.4 ± 8.05 vs. 41.3 ± 8.36	46.9% vs. 78.1%	49.07 ± 8.71 vs. 46.96 ± 7.32	12 months
Abosayed et al. 2022 [17]	Egypt	Obesity Surgery	RCT	91	46	45	37.4 ± 10.2 vs. 34.5 ± 10.7	76.1% vs. 88.9%	46.5 ± 3.8 vs. 46.9 ± 5.8	12 months
Afaneh et al. 2015 [18]	USA	Surgical Endoscopy	RCT	60	30	30	37 ± 9.8 vs. 43 ± 12.6	NR	49.1 ± 8.3 vs. 45.1 ± 7.1	10.7 vs. 7.5 months
Elghandour et al. 2021 [19]	Egypt	Ain-Shams Journal of Surgery	RCT	119	59	60	37.66 ± 11.28 vs. 39.71 ± 11.71	78.0% vs. 66.7%	46.32 ± 6.69 vs. 45.5 ± 7.58	12 months
Ibrahim et al. 2024 [20]	Egypt	World Journal of Laparoscopic Surgery	RCT	376	176	200	35 ± 12 vs. 36 ± 10	43.8% vs. 44.5%	43 ± 9 vs. 44 ± 8	6 months
Khalifa et al. 2018 [21]	Egypt	Bariatric Surgical Practice and Patient Care	RCT	40	20	20	37 ± 8 vs. 30.3 ± 8.4	87.5% vs. 87.5%	49.6 ± 7.3 vs. 43.9 ± 5.2	3 months
Labib et al. 2020 [22]	Egypt	The Egyptian Journal of Hospital Medicine	RCT	172	86	86	39 ± 9.1 vs. 41.3 ± 5.09	67.9% vs. 66.3%	43.7 ± 3.22 vs. 45.58 ± 3.44	16 months
Pilone et al. 2019 [23]	Italy	BMC Surgery	RCT	186	90	96	39.6 ± 5.0 vs. 37.4 ± 3.5	64.4% vs. 54.2%	45.7 ± 3.8 vs. 44.6 ± 4.1	17.5 vs. 16.4 months
Sarhan et al. 2021 [24]	Egypt	The Egyptian Journal of Hospital Medicine	RCT	32	16	16	33 ± 7.8 vs. 32.4 ± 8.4	62.5% vs. 56.3%	NR	12 months
Yapalak et al. 2023 [25]	Turkey	Journal of Laparoscopic and Advanced Surgical Techniques	RCT	60	30	30	32.2 vs. 35.7	80.0% vs. 80.0%	44.1 vs. 45.1	12 months

Outcome Synthesis

**Figure 3:**
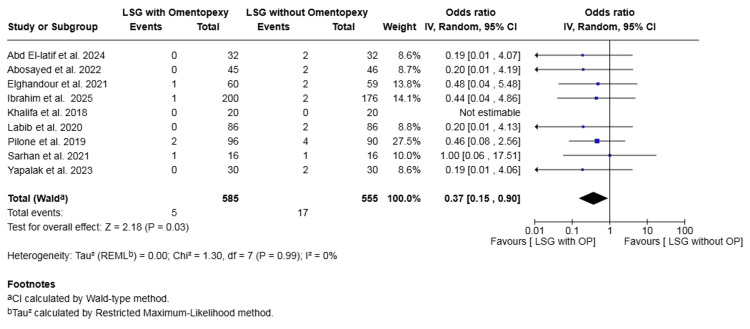
Forest plot comparing LSG with and without omentopexy for the outcome of bleeding LSG: laparoscopic sleeve gastrectomy; OP: omentopexy [16,17,19-25]

Bleeding: Nine studies with a total of 1140 patients reported on bleeding [16,17,19-25]. The rate was 0.85% in the omentopexy group and 3.06% in the group without omentopexy. The omentopexy group was associated with a statistically significant lower risk of bleeding (OR 0.37, 95% CI 0.15 to 0.90, P = 0.03). No heterogeneity was observed across the included studies (I² = 0%, P = 0.99) (Figure 3).

**Figure 4:**
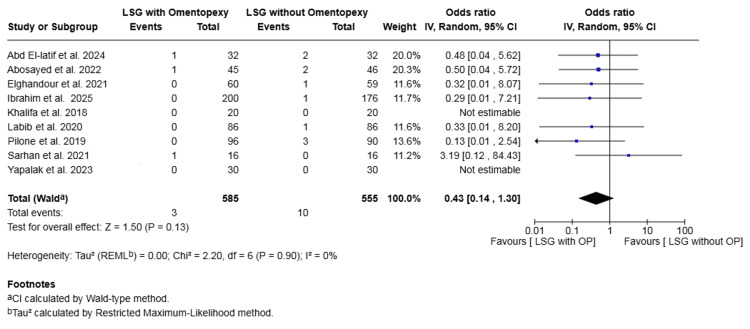
Forest plot comparing LSG with and without omentopexy for the outcome of leakage LSG: laparoscopic sleeve gastrectomy; OP: omentopexy [16,17,19-25]

Leakage: Nine studies with a total of 1140 patients reported on leakage [16,17,19-25]. The rate was 0.51% in the omentopexy group and 1.80% in the group without omentopexy. The difference was not statistically significant (OR 0.43, 95% CI 0.14 to 1.30, P = 0.13). No heterogeneity was observed across the included studies (I² = 0%, P = 0.90) (Figure 4).

**Figure 5:**
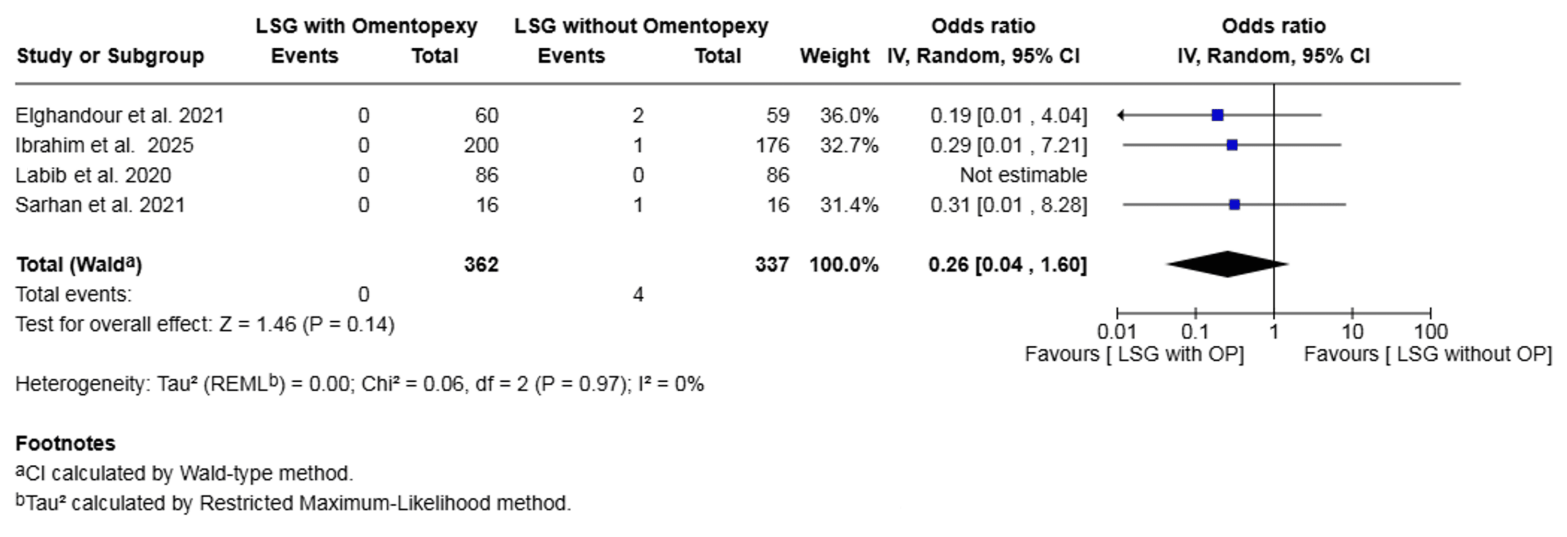
Forest plot comparing LSG with and without omentopexy for the outcome of gastric torsion LSG: laparoscopic sleeve gastrectomy; OP: omentopexy [19,20,22,24]

Gastric torsion: Four studies with a total of 699 patients reported on gastric torsion [19,20,22,24]. The rate was 0.00% in the omentopexy group and 1.19% in the group without omentopexy. The difference was not statistically significant (OR 0.26, 95% CI 0.04 to 1.60, P = 0.14). No heterogeneity was observed across the included studies (I² = 0%, P = 0.97) (Figure 5).

**Figure 6:**
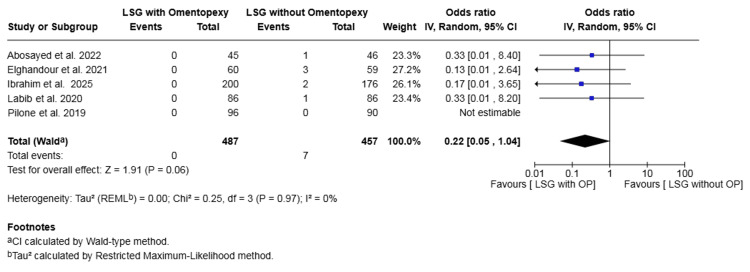
Forest plot comparing LSG with and without omentopexy for the outcome of reintervention LSG: laparoscopic sleeve gastrectomy; OP: omentopexy [17,19,20,22,23]

Reintervention: Five studies with a total of 944 patients reported on reintervention [17,19,20,22,23]. The rate was 0.00% in the omentopexy group and 1.53% in the group without omentopexy. The difference was not statistically significant (OR 0.22, 95% CI 0.05 to 1.04, P = 0.06). No heterogeneity was observed across the included studies (I² = 0%, P = 0.97) (Figure 6).

**Figure 7:**
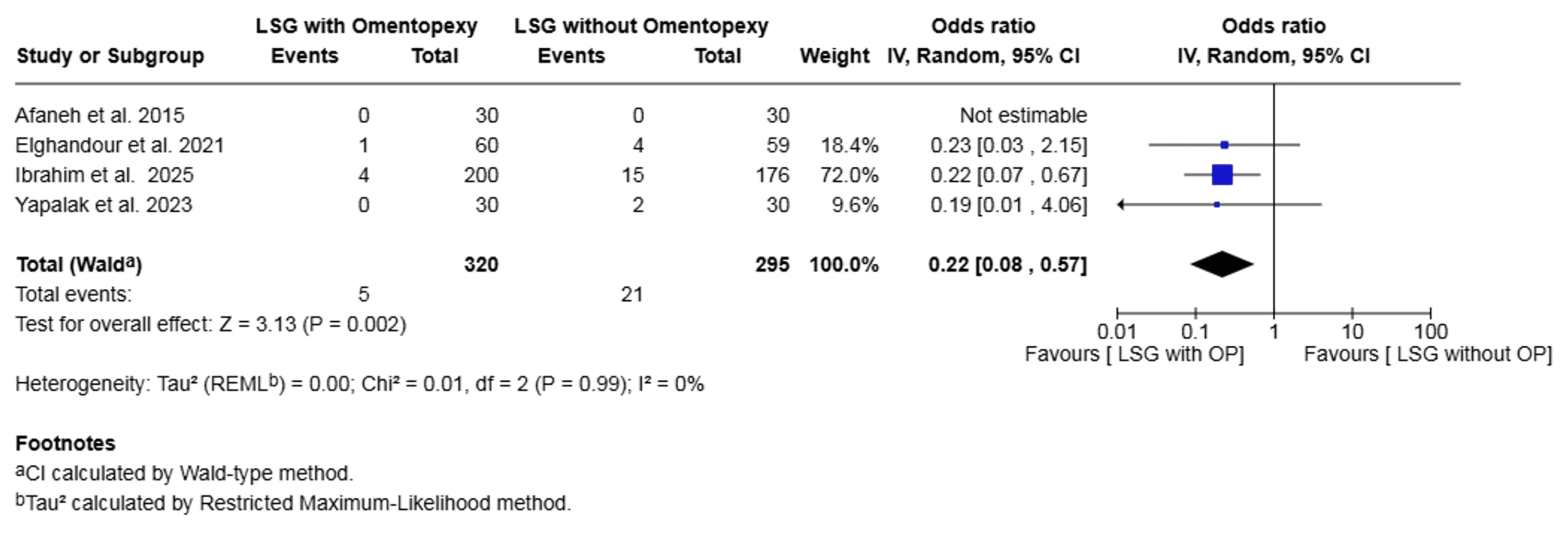
Forest plot comparing LSG with and without omentopexy for the outcome of readmission LSG: laparoscopic sleeve gastrectomy; OP: omentopexy [18-20,25]

Readmission: Four studies with a total of 615 patients reported on readmission [18-20,25]. The rate was 1.56% in the omentopexy group and 7.12% in the non-omentopexy group. The omentopexy group was associated with a statistically significant lower risk of readmission (OR 0.22, 95% CI 0.08 to 0.57, P = 0.002). No heterogeneity was observed across the included studies (I² = 0%, P = 0.99) (Figure 7).

**Figure 8:**
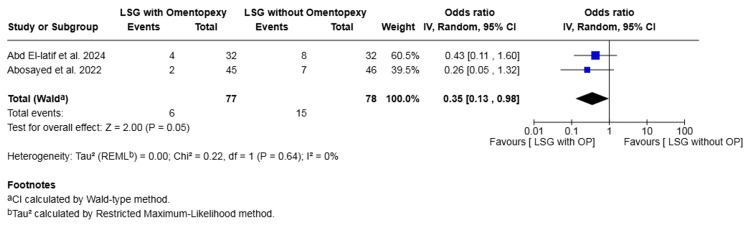
Forest plot comparing LSG with and without omentopexy for the outcome of nausea LSG: laparoscopic sleeve gastrectomy; OP: omentopexy [16,17]

Nausea: Two studies with a total of 155 patients reported on nausea [16,17]. The rate was 7.79% in the omentopexy group and 19.23% in the group without omentopexy. The omentopexy group was associated with a statistically significant lower risk of nausea (OR 0.35, 95% CI 0.13 to 0.98, P = 0.05). No heterogeneity was observed across the included studies (I² = 0%, P = 0.64) (Figure 8). 

**Figure 9:**
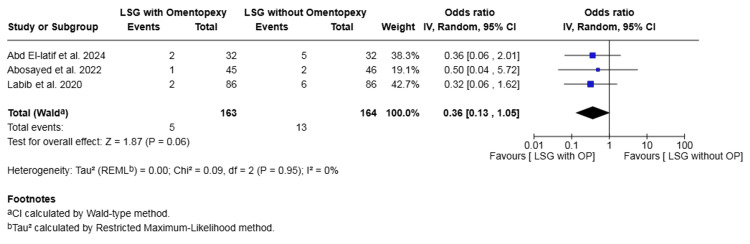
Forest plot comparing LSG with and without omentopexy for the outcome of vomiting LSG: laparoscopic sleeve gastrectomy; OP: omentopexy [16,17,22]

Vomiting: Three studies with a total of 327 patients reported on vomiting [16,17,22]. The rate was 3.07% in the omentopexy group and 7.93% in the group without omentopexy. The difference was not statistically significant (OR 0.36, 95% CI 0.13 to 1.05, P = 0.06). No heterogeneity was observed across the included studies (I² = 0%, P = 0.95) (Figure 9).

**Figure 10:**
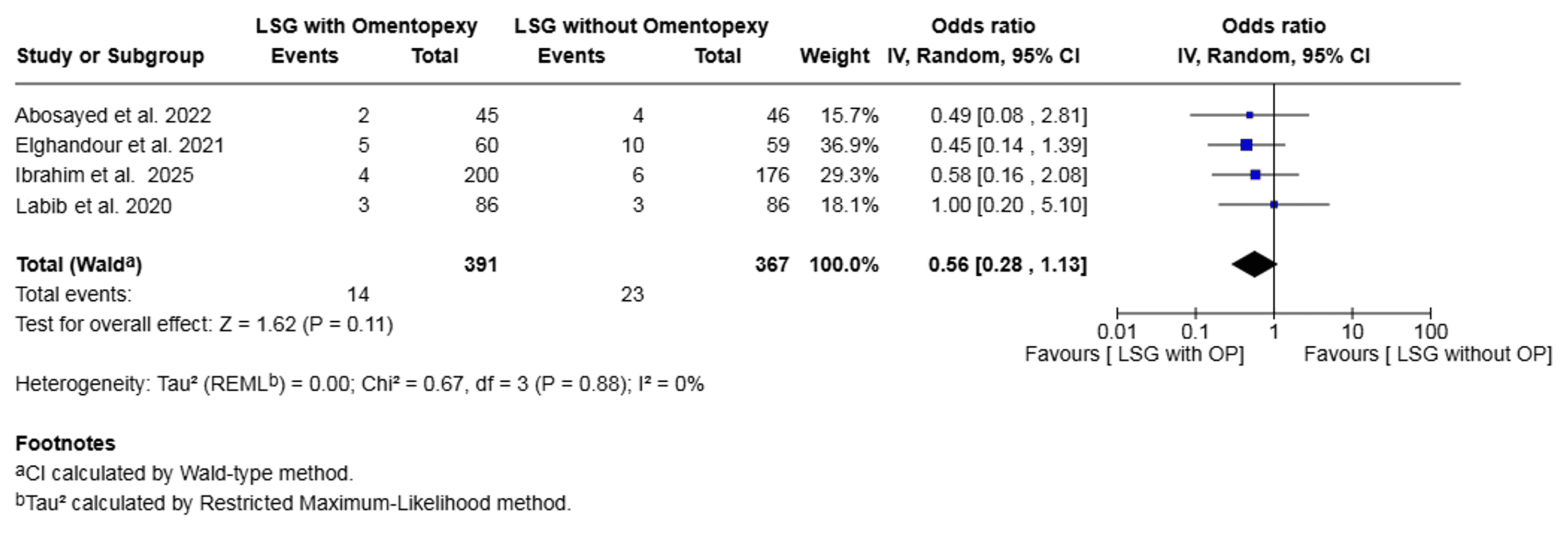
Forest plot comparing LSG with and without omentopexy for the outcome of reflux LSG: laparoscopic sleeve gastrectomy; OP: omentopexy [17,19,20,22]

Reflux: Four studies with a total of 758 patients reported on reflux [17,19,20,22]. The rate was 3.58% in the omentopexy group and 6.27% in the group without omentopexy. The difference was not statistically significant (OR 0.56, 95% CI 0.28 to 1.13, P = 0.11). No heterogeneity was observed across the included studies (I² = 0%, P = 0.88) (Figure 10).

**Figure 11:**
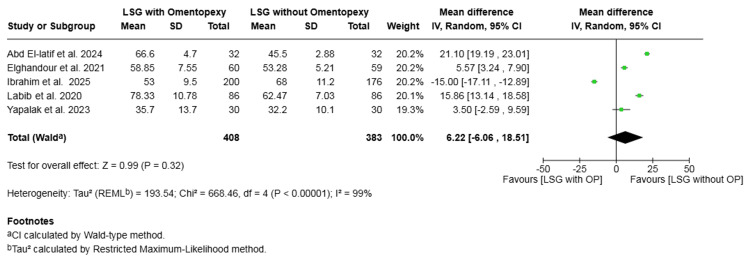
Forest plot comparing LSG with and without omentopexy for the outcome of operative time (minutes) LSG: laparoscopic sleeve gastrectomy; OP: omentopexy [16,19,20,22,25]

Operative time (minutes): Five studies with a total of 791 patients reported on operative time [16,19,20,22,25]. The difference in mean operative time was not statistically significant (mean difference = 6.22, 95% CI -6.06 to 18.51, P = 0.32). Substantial heterogeneity was observed across the included studies (I² = 99%, P < 0.00001) (Figure 11).

**Figure 12:**
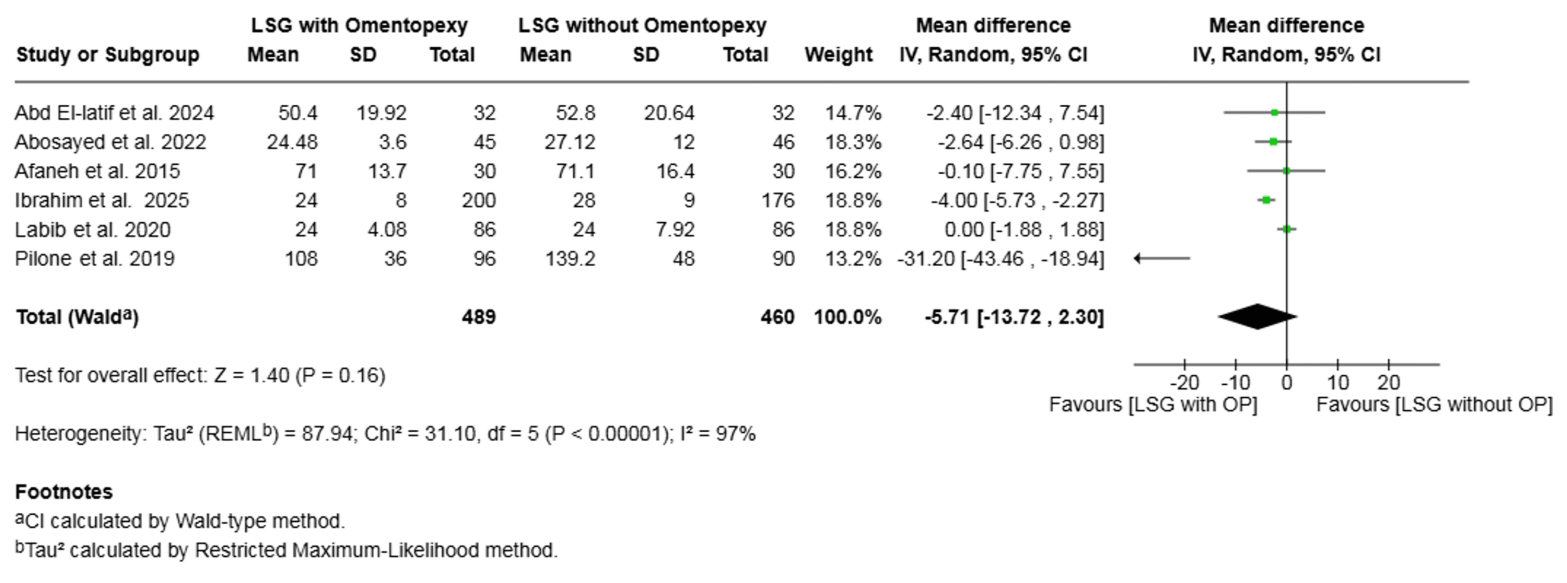
Forest plot comparing LSG with and without omentopexy for the outcome of length of hospital stay (hours) LSG: laparoscopic sleeve gastrectomy; OP: omentopexy [16-18,20,22,23]

Length of hospital stay (hours): Six studies with a total of 949 patients reported on length of hospital stay [16-18,20,22,23]. The difference in mean stay was not statistically significant (mean difference = -5.71, 95% CI -13.72 to 2.30, P = 0.16). Substantial heterogeneity was observed across the included studies (I² = 97%, P < 0.00001) (Figure 12).

**Figure 13:**
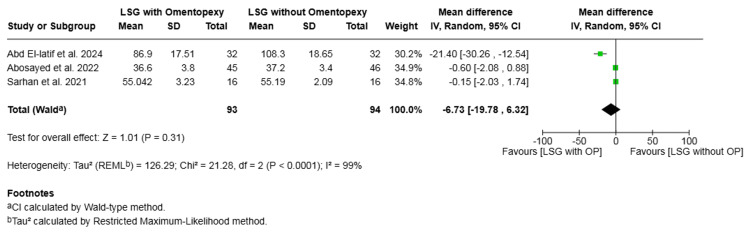
Forest plot comparing LSG with and without omentopexy for the outcome of one-year weight loss LSG: laparoscopic sleeve gastrectomy; OP: omentopexy [16,17,24]

One-year weight loss: Three studies with a total of 187 patients reported on one-year weight loss [16,17,24]. The difference in mean one-year weight loss was not statistically significant (mean difference = -6.73, 95% CI -19.78 to 6.32, P = 0.31). Substantial heterogeneity was observed across the included studies (I² = 99%, P < 0.0001) (Figure 13).

**Figure 14:**
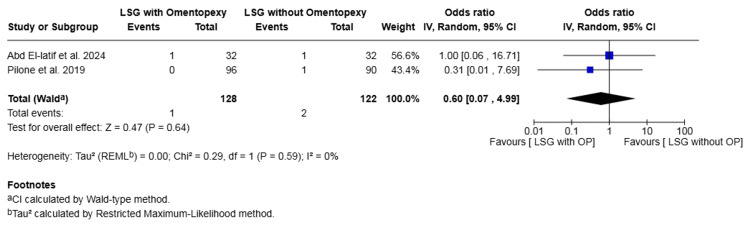
Forest plot comparing LSG with and without omentopexy for the outcome of wound infection LSG: laparoscopic sleeve gastrectomy; OP: omentopexy [16,23]

Wound infection: Two studies with a total of 250 patients reported on wound infection [16,23]. The rate was 0.78% in the omentopexy group and 1.64% in the group without omentopexy. The difference was not statistically significant (OR 0.60, 95% CI 0.07 to 4.99, P = 0.64). No heterogeneity was observed across the included studies (I² = 0%, P = 0.59) (Figure 14).

Heterogeneity Assessment

It is noted that for several dichotomous outcomes (including bleeding, leakage, and torsion), I² was calculated as 0%. Given the low event counts across the included studies, this likely reflects a sparse data bias and the mathematical limitations of random-effects models in rare event settings rather than a definitive absence of clinical heterogeneity.

Sensitivity Analysis

The direction of the result stayed the same whether we used the risk ratio (RR) or the risk difference (RD). We also checked the results by running a leave-one-out analysis. Removing any single study did not change the significance of the final outcome.

Discussion

This meta-analysis demonstrated that the omentopexy group was associated with a significantly lower risk of bleeding, readmission, and postoperative nausea when compared to the non-omentopexy group. No significant differences were found between the two groups with respect to leakage, gastric torsion, reintervention, vomiting, or reflux. The between-study heterogeneity was absent or low for most of the evaluated outcome measures, which indicates the robustness of our findings.

Our findings demonstrate that omentopexy was associated with a significantly lower risk of postoperative bleeding. This may be attributed to the omentum's reconstructive properties and its ability to minimize postoperative bleeding [7]. In contrast, while the observed rate of staple line leakage was more than three times lower in the omentopexy group, this difference did not reach statistical significance. This is supported by several biomechanical principles. After standard LSG, the lateral forces acting on the stomach are lost due to the detachment of the greater omentum, creating imbalanced forces that can lead to kinking and increased intragastric pressure [27,28]. Omentopexy is thought to recreate this lateral stabilization, giving the stomach a smooth, reverse C-shape [11]. Furthermore, the omentum itself acts as a useful tool; it is rich in blood vessels, has a high capacity to absorb fluids, can seal oozing surfaces, and stimulates the growth of new tissue, creating a mechanical barrier that may prevent the spread of digestive fluid [29,30].

Our findings partially diverge from the previous meta-analysis by Zarzycki et al. (2021) [2], which analyzed four studies, of which just one was an RCT. While Zarzycki et al. similarly reported reduced overall morbidity with omental reinforcement, they found a statistically significant reduction in gastric leakage rates (RR: 0.17, P = 0.02). In contrast, our updated analysis - incorporating a significantly larger pool of high-quality evidence (10 RCTs) - did not find a statistically significant difference in leakage (P = 0.13). This distinction suggests that while omentopexy aids in hemostasis (bleeding reduction), its ability to definitively prevent staple line leaks may be less certain when evaluated through strictly randomized evidence.

Our analysis also showed a significant reduction in postoperative nausea for patients receiving omentopexy. Postoperative nausea and vomiting (PONV) is a frequent complication following LSG, potentially caused by intragastric hypertension or damage to the vagus nerve [31]. The stabilization of the gastric remnant by omentopexy may improve gastric emptying and prevent the axial rotation that contributes to these symptoms [31-33]. This is in line with findings from Abdallah et al. (2017) [34], who reported a significantly higher PONV impact in their no omentopexy group. Although the rates of vomiting in our analysis were also lower in the omentopexy group, this difference did not achieve statistical significance.

A key finding of this analysis is the impact of omentopexy on the overall postoperative course, evidenced by a significantly lower risk of readmission. This finding, while statistically significant and consistent across studies, should be interpreted with caution. It is derived from a pooled analysis of only four studies, and the total number of patient events was small. Therefore, the robustness of this estimate is uncertain, and larger trials are needed to confirm this benefit. Furthermore, the need for reintervention trended toward being lower in the omentopexy group, although this did not reach the threshold for statistical significance.

The impact of omentopexy on gastroesophageal reflux disease (GERD) remains controversial. While theoretically stabilizing the Angle of His to mitigate reflux [32,33], our analysis found no statistically significant difference between groups. Although some studies report improved clinical scores [10], our findings align with cohorts showing no reduction in de novo GERD [35]. We suggest this lack of significance may be influenced by the routine postoperative administration of proton-pump inhibitors (PPIs), which potentially masks the true physiological effect of the surgical technique [2,6].

We were not able to demonstrate a significant difference in several key procedural and recovery metrics. The addition of omentopexy did not result in a statistically significant increase in operative time, nor did it significantly reduce the length of hospital stay. An unbiased comparison of the length of stay between studies is difficult, as it can be associated with local customs and discharge protocols rather than objective clinical criteria [36,37]. It is important to note that the analyses for operative time and length of stay were associated with substantial heterogeneity. This high degree of inconsistency means the pooled mean differences for these outcomes should be interpreted with caution. This suggests that factors beyond the use of omentopexy, such as surgeon technique or institutional protocols adhering to Enhanced Recovery After Surgery (ERAS) guidelines [38], are the primary drivers of these outcomes. However, this level of heterogeneity is also a common methodological finding when pooling continuous data from a small number of studies.

The current study has some limitations that should be considered when interpreting its findings. A primary limitation is that omentopexy is not a standardized procedure, with the surgical technique varying among the included studies. This procedural variability likely contributed to the heterogeneity observed in some outcomes, such as operative time. Consequently, the high statistical heterogeneity observed in continuous outcomes necessitates that these pooled estimates be interpreted with caution, as they likely reflect variations in institutional protocols rather than the intervention itself. Geographically, a significant proportion of the included RCTs originated from a single region (Egypt), which may limit the global generalizability of the findings due to specific population characteristics or local healthcare practices. In contrast, for other outcomes like bleeding, the analysis showed zero heterogeneity. This must be interpreted with caution. Given the trials are small, were conducted in different countries, and likely used non-identical omentopexy techniques, it is highly unlikely that there is true zero heterogeneity. The 0% value is likely due to three main reasons. First, most studies have low event counts. This can cause a sparse data bias, which gives a misleading impression of homogeneity. Second, when event rates are low, random-effects models are known to underestimate the true level of heterogeneity. Third, the use of continuity corrections, which can be required for studies with zero events, may also artificially make the data appear more homogenous. Furthermore, the methodological quality of the included trials was variable, and potential confounding from unmeasured variables such as surgeon experience could not be accounted for. We were also unable to perform subgroup analyses based on surgical technique or patient demographics, which may have masked more nuanced differences between the groups. Finally, with a mean follow-up of only 8.8 months, we did not evaluate long-term outcomes such as sustained weight loss or the incidence of de novo GERD.

## Conclusions

The meta-analysis of best available evidence (level 1a) indicates that omentopexy during LSG is associated with a significant reduction in postoperative bleeding, nausea, and readmission rates. These findings might support the use of omentopexy to improve short-term outcomes following sleeve gastrectomy. Nonetheless, the absence of long-term data and evident variability in the omentopexy technique warrant further investigation before routine adoption.

## Appendices

Appendix A

**Table 2:** Search strategy MeSH: medical subject headings; TI: title; AB: abstract; KW: keyword

Search No.	Search Strategy*
#1	MeSH descriptor: [Gastrectomy] explode all trees
#2	"sleeve gastrectomy" OR "laparoscopic sleeve gastrectomy" OR "LSG" OR "vertical sleeve gastrectomy": TI, AB, KW
#3	#1 OR #2
#4	MeSH descriptor: [Omentum] explode all trees
#5	"omentopexy" OR "omento fixation" OR "gastropexy": TI, AB, KW
#6	#4 OR #5
#7	MeSH descriptor: [Postoperative Complications] explode all trees
#8	"outcomes" OR "complications" OR "leak" OR "bleeding" OR "stenosis" OR "nausea" OR "vomiting" OR "staple line": TI, AB, KW
#9	#7 OR #8
#10	MeSH descriptor: [Bariatric Surgery] explode all trees
#11	"bariatric surgery" OR "weight loss surgery": TI, AB, KW
#12	#10 OR #11
#13	#3 AND (#6 OR #9) AND #12
*This search strategy was adopted for the following databases: MEDLINE, Embase, the Cumulative Index to Nursing and Allied Health Literature (CINAHL), the Cochrane Central Register of Controlled Trials (CENTRAL), and Web of Science.	
